# Supplementary figures and images for: The impact of assisted reproductive technologies versus natural conception on neonatal intensive care unit admission: A retrospective cohort analysis
Source: PLoS One. 2025 Sep 2;20(9):e0329943. doi: 10.1371/journal.pone.0329943 (PMC12404392; doi:10.1371/journal.pone.0329943)

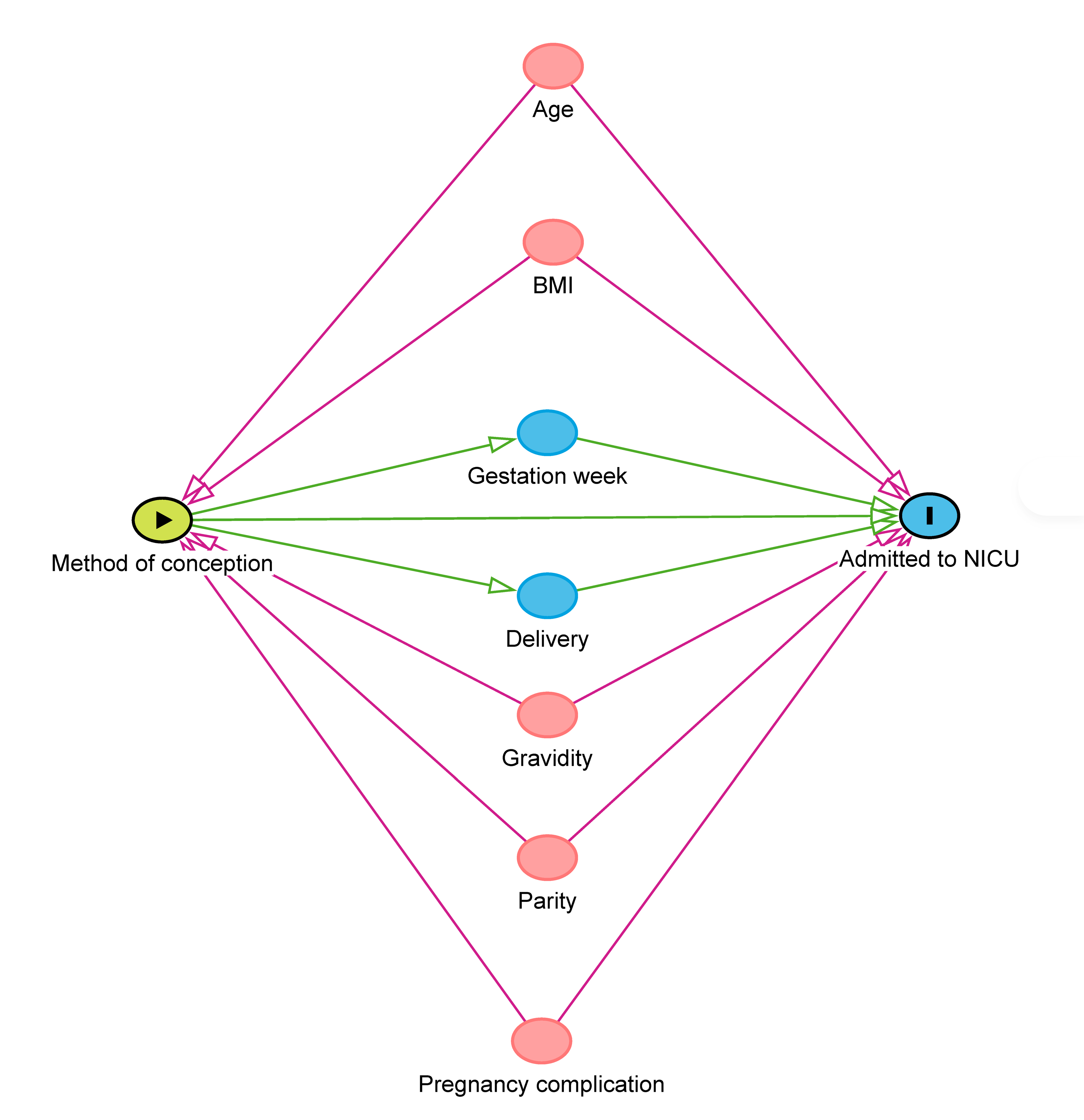

Supplement: S2 Fig — (TIF) [file pone.0329943.s002.tif]
