# Supplementary material for: The impact of assisted reproductive technologies versus natural conception on neonatal intensive care unit admission: A retrospective cohort analysis
Source: PLoS One. 2025 Sep 2;20(9):e0329943. doi: 10.1371/journal.pone.0329943 (PMC12404392; doi:10.1371/journal.pone.0329943)
Supplement: S3 Table — (DOCX) [file pone.0329943.s003.docx]

| **Variable** | **First round of PSM** | | **Second round of PSM** | |
| --- | --- | --- | --- | --- |
| **SMDs** | **Before PSM** | **After PSM** | **Before PSM** | **After PSM** |
| Age | 0.496 | 0.029 | 0.496 | 0.002 |
| BMI | 0.156 | 0.010 | 0.156 | -0.041 |
| Gestation week | -0.150 | 0.026 |  |  |
| Gravidity |  |  |  |  |
| ≤2 | -0.050 | -0.022 | -0.050 | 0.030 |
| ＞2 | 0.050 | 0.022 | 0.050 | -0.030 |
| Parity |  |  |  |  |
| Primipara | 0.213 | 0.039 | 0.213 | 0.077 |
| Multipara | -0.213 | -0.039 | -0.213 | -0.077 |
| Pregnancy complication |  |  |  |  |
| No | 0.030 | -0.012 | 0.030 | -0.015 |
| Yes | -0.030 | 0.012 | -0.030 | 0.015 |
| Delivery |  |  |  |  |
| Cesarean Section | 0.536 | 0.006 |  |  |
| Vaginal Delivery | -0.536 | -0.006 |  |  |
