# Supplementary material for: The impact of assisted reproductive technologies versus natural conception on neonatal intensive care unit admission: A retrospective cohort analysis
Source: PLoS One. 2025 Sep 2;20(9):e0329943. doi: 10.1371/journal.pone.0329943 (PMC12404392; doi:10.1371/journal.pone.0329943)
Supplement: Attachment — Submitted filename: PONE-D-25-15056 Comment.docx [file pone.0329943.s004.docx]

**PONE-D-25-15056**

**Title: The Impact of Assisted Reproductive Technologies versus Natural Conception on Neonatal Intensive Care Unit Admission: A Retrospective Cohort Analysis**

**Comment**: This manuscript addresses an important and timely topic with clinical relevance. The use of PSM, logistic regression models, and subgroup/mediation analyses is statistically sound and strengthens the findings. The key conclusion—that ART-conceived neonates may have a lower risk of NICU admission compared to naturally conceived neonates—contrasts with existing literature, making it a notable contribution. The manuscript holds promise but currently needs **major revisions** before it can be considered for publication. However, some revisions are necessary before publication:

**Comment 1**: The study's finding that ART-conceived neonates have a lower NICU admission risk is highly unexpected and largely contradicts existing literature. The authors' explanations (e.g., enhanced prenatal monitoring, eSET) are speculative and lack direct empirical support within the study. Furthermore, their discussion's claim of "partial control of confounding factors" undermines the stated effectiveness of their PSM methodology in balancing these factors. A more thorough, data-driven exploration of this significant discrepancy is needed.

**Comment 2**: The data collection period (January 2022 to December 2024) is remarkably recent for a retrospective study being submitted in June 2025. This raises questions about the completeness of data extraction and validation within such a tight timeframe. Retrospective studies typically involve data from past years to ensure all outcomes have occurred and records are finalized.

**Comment 3:** While PSM helps, retrospective studies are inherently susceptible to selection bias. Factors leading to ART are complex, and even with matching, unmeasured confounders (e.g., specific ART protocols, underlying subfertility causes, pre-existing maternal health conditions not captured as "complications," lifestyle factors beyond what was measured) could still influence outcomes and bias the results.

**Comment 4**: While broad categories like "pregnancy complication" are mentioned, more specific details on the types and severity of these complications (e.g., pre-eclampsia vs. gestational hypertension, controlled vs. uncontrolled diabetes) could strengthen the analysis, as these can significantly impact NICU admission. The manuscript does not explicitly state the criteria for NICU admission at their hospital. This is important as admission criteria can vary between institutions and influence the outcome measure.

**Comment 5:** The abstract states PSM was used to match "seven potential confounders" and later that "PSM was applied to five maternal factors." The "Methodology" section lists "maternal age, BMI, gestational age, gravidity, parity, gestational comorbidities, mode of conception (natural or ART)" as variables collected. Table 1 shows seven variables used for PSM (Age, BMI, Gestation week, Gravidity, Parity, Pregnancy complication, Delivery). This inconsistency between the abstract and the methods/results needs to be clarified. It is crucial to be precise about which variables were included in the PSM model for each analysis. The "Finally, PSM was applied to five maternal factors (age, BMI, gestational age, number of births, and pregnancy-related comorbidities)" in the abstract seems to refer to a separate PSM run for the box scatter plots. This should be explicitly detailed as a distinct analysis step in the Methods section.

**Comment 6**: The mediation analysis findings, particularly "the proportion mediated was -15.07%" for gestational weeks, are unusual. A negative proportion mediated suggests a suppressor effect, where the mediator (gestational weeks) works in the opposite direction to the direct effect, or it could indicate an unmeasured confounder or complex interaction. This finding needs a more detailed explanation and interpretation in the discussion, rather than just stating the proportion. What are the clinical implications of gestational week acting as a negative mediator?

**Comment 7**: The manuscript is generally well-structured, but there are minor grammatical errors and awkward phrasings that could be improved with careful proofreading. Some statements in the discussion ("assisted vaginal delivery was associated with an increased risk [5.77 (4.01–8.31)]") seem to be results being re-stated without further discussion in the relevant context. The term "assisted vaginal delivery" in Table 2 seems to be contrasted with "Cesarean Section" and "Vaginal Delivery," which is confusing. It appears to refer to vaginal delivery, not specifically assisted vaginal delivery. This requires clarification.
