# Supplementary material for: The impact of assisted reproductive technologies versus natural conception on neonatal intensive care unit admission: A retrospective cohort analysis
Source: PLoS One. 2025 Sep 2;20(9):e0329943. doi: 10.1371/journal.pone.0329943 (PMC12404392; doi:10.1371/journal.pone.0329943)
Supplement: Attachment — Submitted filename: Response to Reviewers.docx [file pone.0329943.s005.docx]

**Response Letter**

Dear editors and reviewers,

We deeply appreciate your valuable suggestions and comments, which enable us to further improve our paper. We have studied the comments carefully and have made our best efforts to revise the entire paper as suggested by the editor and reviewers. We are very pleased that our paper can be revised, some **detailed responses** are as follows.

**To Editors:**

1. Please ensure that your manuscript meets PLOS ONE's style requirements, including those for file naming.

**Response :** Thank you for your guidance regarding PLOS ONE's style requirements. We have carefully reviewed and ensured our manuscript fully complies with the journal's formatting standards, including file naming conventions.

2. PLOS requires an ORCID iD for the corresponding author in Editorial Manager on papers submitted after December 6th, 2016. Please ensure that you have an ORCID iD and that it is validated in Editorial Manager. To do this, go to ‘Update my Information’ (in the upper left-hand corner of the main menu), and click on the Fetch/Validate link next to the ORCID field. This will take you to the ORCID site and allow you to create a new iD or authenticate a pre-existing iD in Editorial Manager.

**Response :** Thank you for your note regarding the ORCID requirement. We confirm that the corresponding author has a valid ORCID iD (0009-0000-0596-6734) and has completed the validation process in Editorial Manager as instructed. The ORCID was successfully linked and authenticated through the 'Fetch/Validate' feature in the 'Update my Information' section, and the status now shows as validated in the system.

3. Your ethics statement should only appear in the Methods section of your manuscript. If your ethics statement is written in any section besides the Methods, please move it to the Methods section and delete it from any other section. Please ensure that your ethics statement is included in your manuscript, as the ethics statement entered into the online submission form will not be published alongside your manuscript.

**Response :** Thank you for your guidance regarding the ethics statement. We confirm that we have moved the ethics statement to the Methods section (subsection [The study population]) and removed it from all other sections of the manuscript.

**To Reviewer #1:**

Abstract
• Strengthen the rationale by briefly explaining why ART pregnancies might result in lower NICU admission rates (e.g., improved prenatal care, selective embryo transfer).
**Response :** We sincerely appreciate your valuable suggestion to strengthen the rationale regarding ART pregnancies and NICU admission rates. In response to this comment, we have added explanatory statements in both the Results and Conclusion sections of the abstract:

In the Results section, we added: "This may be attributed to enhanced prenatal monitoring and selective embryo transfer in ART pregnancies, which could mitigate adverse perinatal outcomes."

In the Conclusion section, we included: "potentially due to optimized prenatal care and embryo selection in ART procedures."

These additions explicitly address the potential mechanisms that may contribute to the observed lower NICU admission rates in ART-conceived neonates. We have marked these additions in red text for easy identification.

Thank you for this constructive suggestion, which has helped improve the clarity and scientific rigor of our abstract.

Introduction
• Clearly state the rationale for the hypothesis that ART may reduce NICU admissions. Discuss relevant mechanisms such as increased prenatal surveillance, use of elective single embryo transfer (eSET), and higher socioeconomic status among ART users.

**Response :** We sincerely appreciate your valuable suggestion regarding the need to clarify the rationale for our hypothesis that ART may reduce NICU admissions. In response, we have added a dedicated paragraph in the Introduction (marked in red) that explicitly outlines the key mechanisms, including: (1) closer prenatal monitoring enabling early complication detection, (2) elective single embryo transfer (eSET) reducing multifetal gestation risks, and (3) higher socioeconomic status among ART users improving healthcare access. We have also added connecting sentences in subsequent paragraphs (also marked in red) to reinforce these concepts throughout the Introduction. These revisions have significantly strengthened our theoretical framework by clearly articulating the potential pathways through which ART might lower NICU admission rates compared to natural conception. We are grateful for this constructive suggestion that has enhanced the clarity and scientific rigor of our manuscript.

Methods
Definition of ART
• Provide a clear definition of what procedures were included under “ART” (e.g., IVF, ICSI, frozen embryo transfer, ovulation induction, IUI).
• If disaggregated data are unavailable, acknowledge this as a limitation.

**Response :** We sincerely appreciate your valuable suggestion regarding the need to clarify our definition of ART procedures. In response to your comment, we have added the following paragraph (marked in red text) to the Data Collection section of our Methods: "In our study, the term ART encompasses the following specific procedures: in vitro fertilization (IVF), intracytoplasmic sperm injection (ICSI), frozen embryo transfer (FET), ovarian induction (OI), intrauterine insemination (IUI), and blastocyst transfer. However, it should be noted that while our dataset includes all these ART modalities, the medical records did not consistently specify the exact type of ART procedure for each case. As a result, our analysis treats ART as a collective variable rather than examining outcomes by specific ART subtypes. We acknowledge this as a methodological limitation that prevents us from conducting more granular analyses of differential outcomes across various ART techniques. This limitation primarily stems from the retrospective nature of our data collection from clinical records." This addition clearly defines the scope of ART procedures included in our study, explicitly acknowledges the limitation of not having disaggregated data by ART type, and provides the rationale for this limitation (retrospective data collection from clinical records). Thank you again for this constructive suggestion that has enhanced the quality of our manuscript.

NICU Admission Criteria
• Describe the clinical or institutional criteria used to define NICU admission. If unavailable, discuss as a limitation affecting generalizability.

**Response :** Thank you for this important methodological consideration. As requested, we have now explicitly detailed the NICU admission criteria in the Data Collection subsection of our Methods section , with the following addition in red font:

"The admission criteria for the NICU in our study followed a standardized institutional protocol that included: (1) perinatal asphyxia; (2) significant birth trauma (e.g., clavicular fracture or intracranial hemorrhage); (3) respiratory disorders requiring support (including pneumonia, pulmonary complications, or mechanical ventilation); (4) confirmed or suspected sepsis; (5) very low birth weight (<1500 g) or gestational age <32 weeks; (6) major congenital anomalies needing immediate intervention (e.g., congenital heart defects); and (7) conditions secondary to intrauterine infection."

This clarification ensures complete transparency regarding our patient selection process and strengthens the methodological rigor of our study. We believe these details will help readers better contextualize our findings.

Propensity Score Matching
• Clarify which covariates were included in each round of PSM (seven in one instance, five in another).
• Justify why a second PSM with fewer variables was conducted.

**Response :** We sincerely appreciate your valuable comments regarding our propensity score matching (PSM) methodology. In response to your request for clarification, we would like to explain our two-stage PSM approach:

Primary PSM Analysis (7 covariates):

This initial matching, used for our main study outcomes, included the following covariates: maternal age, BMI, gestational age, parity, gravidity, pregnancy comorbidities, and delivery mode. These variables were selected as they represent known potential confounders in the relationship between ART conception and NICU admission.

Secondary PSM Analysis (5 covariates):

The subsequent matching focused specifically on five relatively stable maternal characteristics (age, BMI, gravidity, parity, and pregnancy comorbidities) that are typically established earlier in pregnancy. This secondary analysis served two purposes: (1) To generate comparative box plots demonstrating the distribution of more variable outcomes (gestational weeks, birth weights, delivery hemorrhage) before and after matching. (2) To assess the robustness of our findings using a more conservative set of baseline characteristics. This clarification has been added to the Statistical Analysis section (red-highlighted), and we are grateful for your expert guidance which improved our methodological transparency.

• Include a table of standardized mean differences (SMDs) before and after matching.

**Response :** We sincerely appreciate your suggestion regarding the presentation of standardized mean differences (SMDs) and would like to clarify that while we have currently presented the SMD results graphically in Figures 2B for intuitive visualization of balance improvement across all variables (with all post-PSM SMDs <0.1 clearly demonstrated), we fully recognize the value of providing the complete numerical data and would be pleased to incorporate the full SMD table as Supplemental Table SMD (**S3**), which would include all values from both PSM rounds (showing for example how age improved from SMD 0.496 to 0.029 in first round and 0.002 in second round, BMI from 0.156 to 0.010 and -0.041 respectively, and cesarean section from 0.536 to 0.006 in the first round), while maintaining the current figures for visual impact, as we believe this combined approach will maximize both accessibility of key results and completeness of methodological reporting, and we thank you for this valuable suggestion that will enhance the transparency of our matching results.

| **Variable** | **First round of PSM** | | **Second round of PSM** | |
| --- | --- | --- | --- | --- |
| **SMDs** | **Before PSM** | **After PSM** | **Before PSM** | **After PSM** |
| Age | 0.496 | 0.029 | 0.496 | 0.002 |
| BMI | 0.156 | 0.010 | 0.156 | -0.041 |
| Gestation week | -0.150 | 0.026 |  |  |
| Gravidity |  |  |  |  |
| ≤2 | -0.050 | -0.022 | -0.050 | 0.030 |
| ＞2 | 0.050 | 0.022 | 0.050 | -0.030 |
| Parity |  |  |  |  |
| Primipara | 0.213 | 0.039 | 0.213 | 0.077 |
| Multipara | -0.213 | -0.039 | -0.213 | -0.077 |
| Pregnancy complication |  |  |  |  |
| No | 0.030 | -0.012 | 0.030 | -0.015 |
| Yes | -0.030 | 0.012 | -0.030 | 0.015 |
| Delivery |  |  |  |  |
| Cesarean Section | 0.536 | 0.006 |  |  |
| Vaginal Delivery | -0.536 | -0.006 |  |  |

Mediation Analysis
• Add a directed acyclic graph (DAG) to clarify assumptions.

**Response :** We sincerely appreciate the reviewer's valuable suggestion regarding the mediation analysis. In response to this comment, we have carefully constructed and included a directed acyclic graph (DAG) as Supplementary Table DAG (**S2**) to explicitly clarify our causal assumptions and the relationships between variables in the mediation model. We have also added a brief description of the DAG in the Methods section (marked in red text). We are grateful for this suggestion which has improved the clarity of our causal inference framework.

• Discuss potential biases, such as collider stratification, especially when mediators (e.g., gestational age) may also act as confounders.

**Response :** We sincerely thank you for raising this important methodological consideration regarding potential collider stratification bias in our mediation analysis. In response, we have carefully addressed this issue by expanding the discussion in our Methods section's statistical analysis subsection (marked in red font) to examine gestational age and delivery mode may serve dual roles as both mediators and confounders in our analytical framework. We have incorporated a discussion of the potential for collider bias when adjusting for variables that are simultaneously influenced by both the exposure and other unmeasured factors. We fully agree with you that this represents a critical consideration for our mediation analysis, and we believe these additions significantly enhance the methodological rigor and transparency of our study by providing readers with a more comprehensive understanding of both our analytical approach and its inherent limitations. We are grateful for this constructive suggestion which has substantially improved our manuscript.

• Specify the method used for mediation analysis.

**Response :** We sincerely appreciate your comment regarding the specification of our mediation analysis method. In response to this valuable suggestion, we have explicitly stated in the Methods section (highlighted in red font) that we employed a Bayesian mediation analysis approach. We are grateful for this comment which has strengthened the completeness of our methods reporting.

Results
Data Presentation
• Simplify or consolidate figures (e.g., combine boxplots for birthweight, gestational age, and hemorrhage).

**Response :** We sincerely appreciate your suggestion to consolidate figures for improved clarity. However, after careful consideration, we found that combining the boxplots for birth weight, gestational age, and delivery hemorrhage into a single figure would compromise interpretability because:

Different Measurement Scales : Birth weight (grams) and gestational age (weeks) use vastly different numerical ranges (e.g., 2000–4000 g vs. 28–42 weeks). Delivery hemorrhage (mL) has an even wider scale (e.g., 100–1000 mL).

To enhance clarity while adhering to statistical standards, we standardized the formatting across all boxplots with consistent colors, spacing, and annotations. We remain grateful for your valuable feedback.

• Ensure all figure legends are sufficiently detailed for interpretation.

**Response :** We sincerely appreciate your valuable suggestion regarding figure legends. In response to your comment, we have carefully reviewed and revised all figure legends in the manuscript to ensure they now include clear descriptions of each panel's content, definitions of all abbreviations and symbols used, explanations of statistical representations , We believe these enhanced legends will significantly improve readers' ability to interpret our figures independently, and we thank you for this constructive suggestion which has strengthened the clarity of our data presentation. The revised figure legends are included in the updated manuscript.

Subgroup and Mediation Interpretation
• Elaborate on why significant interactions (e.g., with cesarean section) were observed before PSM but not after.

**Response :** We sincerely appreciate your insightful question regarding the differential interaction effects observed before and after propensity score matching (PSM). The attenuation of significant interactions (particularly for cesarean section) post-PSM can be explained by two key methodological and clinical factors:

Confounding Control: The pre-PSM analysis likely reflected confounding by indication, where cesarean sections were disproportionately performed in higher-risk ART pregnancies (e.g., older mothers, multiple gestations). PSM balanced these baseline characteristics, isolating the true mediation effect.

Sample Size Reduction: PSM necessarily reduces sample size (n=3867 pre-PSM → n=2140 post-PSM), decreasing power to detect interaction effects. The wider confidence intervals post-PSM reflect this.

We have added this interpretation to the Results section (marked in red). Thank you for prompting this important clarification.

• Provide clearer interpretation of mediation effect size and its clinical implications.

**Response :** We sincerely appreciate your valuable suggestion regarding the interpretation of our mediation analysis results. In response to your comment, we have enhanced the clinical and mechanistic interpretation by clarifying that: (1) the 18.98% mediation through delivery mode reflects both procedural advantages of cesarean delivery and intensified perinatal monitoring in ART pregnancies, accounting for approximately one-fifth of ART's protective effect; (2) the -15.07% mediation by gestational age represents a suppressor effect where ART embryo selection may compensate for risks typically associated with shorter gestation; and (3) the majority (≈80%) of ART's protective effect operates through other pathways, with important implications for obstetric management. We have added this enhanced interpretation to the "Mediation effect analysis" section of our revised manuscript, with the new content marked in red font for easy identification. Thank you again for your valuable suggestion that has helped improve the clarity and clinical relevance of our findings.

Discussion
• Avoid overstating the significance of effect sizes (e.g., OR ~0.65). Emphasize the modest magnitude of association.

**Response :** We sincerely appreciate your insightful comment regarding the interpretation of our effect sizes. In response to this valuable suggestion, we have carefully revised the Discussion section to: (1) explicitly acknowledge the modest magnitude of the observed associations (e.g., OR ~0.65), (2) remove any language that could be construed as overstating the clinical significance of these effects, and (3) emphasize the need for cautious interpretation given the effect sizes. We agree with you that proper characterization of effect sizes is crucial for appropriate clinical interpretation of our findings, and we are grateful for this suggestion which has improved the precision and balance of our work.

• Expand discussion of limitations, including:
o Lack of ART subtype stratification,
o Single-center design and possible selection bias,
o Unmeasured confounders (e.g., maternal health behaviors, socioeconomic factors).

**Response :** We sincerely appreciate your valuable suggestions for improving our discussion of study limitations. In response, we have thoroughly revised the final paragraph of the Discussion section (marked in red font) to provide a more comprehensive analysis of the study's constraints. We have specifically expanded the discussion to include: (1) detailed consideration of the lack of ART subtype stratification (e.g., IVF vs. ICSI, fresh vs. frozen embryo transfer) and its potential impact on results; (2) an in-depth examination of possible selection bias arising from the single-center design, including its implications for generalizability; and (3) systematic discussion of important unmeasured confounders such as maternal health behaviors (nutrition, exercise, substance use) and socioeconomic factors (education, income, healthcare access). These additions have been carefully integrated into the existing limitations paragraph while maintaining the original discussion of PSM strengths, short-term outcome limitations, and need for future research. We are grateful for your insightful comments which have helped strengthen our work.

Conclusion
• Add a note of caution regarding the generalizability of findings and the limited clinical significance of the observed effect sizes.
• Suggest directions for future research (e.g., multi-center validation, stratified ART analysis, long-term outcomes).

**Response :** We sincerely appreciate your constructive suggestions for improving the conclusion of our manuscript. In response, we have carefully revised the conclusion to incorporate (1) a note of caution regarding the clinical significance and generalizability of our findings, explicitly stating that while the results are statistically significant, their clinical relevance requires further validation, and (2) specific recommendations for future research directions, including the need for multicenter studies with long-term follow-up to validate and extend our observations. These additions have been seamlessly integrated into the existing conclusion to maintain clarity and flow while addressing the reviewer’s important points. We believe these revisions have strengthened the manuscript by providing a more balanced and comprehensive interpretation of our findings, and we are grateful for your insightful comments, which have helped improve the quality of our work.

Language and Style
• Revise terminology for clarity and accuracy (e.g., “delivery hemorrhage” → “intrapartum hemorrhage”).

**Response :** We sincerely apologize for the imprecise terminology used in our original manuscript and greatly appreciate your careful attention to this important detail. In response to this comment, we have systematically revised all instances of "delivery hemorrhage" to the more accurate "intrapartum hemorrhage" throughout the manuscript, with these changes highlighted in red font for easy identification. We appreciate the opportunity to correct this oversight and strengthen our manuscript.

• Consider professional English editing to improve grammar and consistency.

**Response :** We sincerely appreciate your valuable suggestions regarding language refinement. In response, we have carefully revised the manuscript to improve grammar, syntax, and stylistic consistency. These revisions enhance the manuscript's clarity and readability while strictly preserving its original scientific content and meaning. Any language deficiencies in the initial submission are regretted, and we are grateful for your recommendations, which have significantly improved the manuscript's overall quality. All revisions have been systematically implemented, and we believe the current version meets the journal's high linguistic standards.

References
• Update with recent meta-analyses (2021–2024) or large-scale cohort studies related to ART and neonatal outcomes.

**Response :** We sincerely appreciate your valuable suggestion to strengthen our references with recent meta-analyses and large-scale cohort studies. In response, we have updated references 13, 14, 15, 16, and 18 with the most current and high-quality evidence (2021-2024) on ART and neonatal outcomes. Below are the key updates made:

**13.** Smeltzer, S, Acharya, K, Truong, T, Pieper, C, Muasher, S. Clinical pregnancy (CP) and live birth (LB) increase significantly with each additional fertilized oocyte up to nine, and CP and LB decline after that: an analysis of 15,803 first fresh in vitro fertilization cycles from the Society for Assisted Reproductive Technology registry. FERTIL STERIL. 2019-09-01; 112 (3): 520-526.e1. doi: 10.1016/j.fertnstert.2019.04.023. PMID: 31227285

**14.** Zhang, X, Chambers, GM, Venetis, C, Choi, SKY, Gerstl, B, Ng, CHM, Abbott, JA. Perinatal and infant outcomes after assisted reproductive technology treatment for endometriosis alone compared with other causes of infertility: a data linkage cohort study. Fertil Steril. 2025-05-01; 123 (5): 846-855. doi: 10.1016/j.fertnstert.2024.12.007. PMID: 39672363

**15.** Ginström Ernstad, E, Spangmose, AL, Opdahl, S, Henningsen, AA, Romundstad, LB, Tiitinen, A, Gissler, M, Wennerholm, UB, Pinborg, A, Bergh, C, Malchau, SS. Perinatal and maternal outcome after vitrification of blastocysts: a Nordic study in singletons from the CoNARTaS group. HUM REPROD. 2019-11-01; 34 (11): 2282-2289. doi: 10.1093/humrep/dez212. PMID: 31687765

**16.** Wei, D, Liu, JY, Sun, Y, Shi, Y, Zhang, B, Liu, JQ, Tan, J, Liang, X, Cao, Y, Wang, Z, Qin, Y, Zhao, H, Zhou, Y, Ren, H, Hao, G, Ling, X, Zhao, J, Zhang, Y, Qi, X, Zhang, L, Deng, X, Chen, X, Zhu, Y, Wang, X, Tian, LF, Lv, Q, Ma, X, Zhang, H, Legro, RS, Chen, ZJ. Frozen versus fresh single blastocyst transfer in ovulatory women: a multicentre, randomised controlled trial. LANCET. 2019-03-30; 393 (10178): 1310-1318. doi: 10.1016/S0140-6736(18)32843-5. PMID: 30827784

**18.** Wang, L, Xie, J, Chu, Y, Chen, J, Jin, L, Yue, J. Effect of letrozole cotreatment in progestin-primed ovarian stimulation on IVF/ICSI outcomes in POSEIDON group 3 and 4 poor responders: a retrospective cohort study. Eur J Med Res. 2025-04-22; 30 (1): 314. doi: 10.1186/s40001-025-02393-x. PMID: 40259429

Thank you for this constructive suggestion to improve our manuscript.

Transparency and Author Contributions
• Add a data curation statement describing who had access to the dataset and was responsible for its analysis and integrity.

**Response :** We sincerely appreciate your valuable suggestion regarding data transparency. In response to your comment, we have added a dedicated "Data Curation Statement" at the end of the manuscript. We are grateful for this suggestion which has strengthened the methodological rigor of our manuscript.

The statement reads:

"The dataset used in this study was managed and curated under strict protocols to ensure data integrity and security. Access to the original dataset was restricted to the corresponding author, who performed all data cleaning, validation, and preprocessing steps. All analytical procedures were conducted by [First Author], with independent verification performed by [Corresponding Author] to ensure reproducibility. Any data-related inquiries may be directed to the corresponding author."

**To Reviewer #2:**

ABSTRACT
-Suggest removing “in recent years” from “has become a critical issue in perinatal medicine in recent years”. It could be perceived as redundant, the author has made the case for recent & increasing use of ART.

**Response :** We sincerely appreciate your careful reading and constructive suggestion regarding the phrasing in our abstract. In response to your comment, we have removed the phrase "in recent years" from the sentence in question. The revised text now reads:

"Whether these reproductive methods impact neonatal health, particularly by increasing the risk of neonatal intensive care unit (NICU) admission, has become a critical issue in perinatal medicine."

This modification has helped tighten our language while maintaining the full meaning of the statement. Thank you for this thoughtful suggestion that has improved the conciseness of our abstract. We appreciate your attention to detail in helping us enhance the quality of our manuscript.

-Would recommend revising the methods section to condense descriptions of analyses. I think that these could be presented more concisely. For example---“further investigation was carried out through restricted multi-model logistic regression analyses”. It may be difficult for those unfamiliar with the analyses to know what that means or gain significant insight into the project according to that statement.
**Response :** We sincerely appreciate your insightful suggestions regarding methodological clarity. We have thoroughly revised the Methods section to enhance conciseness while maintaining scientific rigor. For example:

1. Replaced technical phrases like "restricted multi-model logistic regression analyses" with more accessible terminology:

Original: "Further investigation was carried out through restricted multi-model logistic regression analyses"

Revised: "with additional multivariable-adjusted models for deeper investigation"

2. Consolidated repetitive references to PSM timepoints (now uniformly using "pre-/post-PSM").

3. Maintained all critical elements (PSM covariates, subgroup stratification, mediation analysis) while reducing redundancy.

4. Adopted parallel structure for analytical steps (e.g., consistent verb tense and phrasing).

These changes improve accessibility for interdisciplinary readers without compromising methodological transparency. Thank you again for this constructive suggestion that has enhanced the quality of our manuscript.

INTRODUCTION
-The following sentence feels out of place in the paragraph--- “Concurrently, cesarean section rates remain high worldwide”.

**Response :** We sincerely appreciate your valuable suggestion regarding the flow of our introduction. In response to your comment, we have removed the sentence "Concurrently, cesarean section rates remain high worldwide" from the introductory paragraph as it was indeed somewhat disconnected from the immediate context discussing ART trends. This revision has helped maintain better focus on the core theme of ART and neonatal outcomes while improving the logical progression from NICU importance to rising ART use and its potential impacts. Thank you for this constructive suggestion which has enhanced the clarity and cohesion of our introduction.

-If the primary difference between this study and others that have investigated this question is the use of propensity score matching, I would recommend including some additional information in the introduction about what makes propensity score matching well suited to this question.

**Response :** We sincerely appreciate your insightful suggestion regarding the need to better highlight the methodological strengths of our study. In response to your valuable comment, we have made the following substantive additions to the Introduction (marked in red text for easy identification):

In the third paragraph, we added:

"These discrepancies may stem from variations in study design, sample size, and inadequate control for key confounding factors such as maternal age, parity, and pre-existing conditions. Propensity score matching (PSM) is particularly well-suited to address these limitations as it: (1) balances observed covariates between ART and naturally conceived groups, (2) mimics randomization in observational studies by creating comparable cohorts, and (3) allows simultaneous adjustment for multiple potential confounders that may differentially affect NICU admission risk.

In the fourth paragraph, we enhanced:

"Unlike previous studies, we used PSM to rigorously control for seven key maternal and obstetric confounders, thereby reducing selection bias and enabling more accurate estimation of the true association between conception method and NICU admission risk."

These modifications explicitly explain why PSM is methodologically advantageous for studying ART-NICU associations and detail our specific application of PSM in this study. Thank you for this constructive suggestion which has greatly improved our manuscript.

-I would recommend including a clear purpose or objective statement at the end of the introduction.

-It seems like there are primary (ART & NICU Admission) and secondary (ART + other outcomes---c/s?) objectives. It would be helpful to clearly state these at the end of the introduction.

**Response :** We sincerely appreciate your valuable suggestion regarding the need for a clearer statement of purpose. In response to your comment, we have added an explicit objective statement at the end of the introduction (marked in red text for easy identification):

"The primary objective of this study was to determine whether ART conception is associated with differential risk of NICU admission compared to natural conception, while controlling for potential confounding factors through propensity score matching. Additionally, we aimed to explore potential mediating factors that might explain any observed associations."

These additions clearly articulate the study's primary and secondary objectives. Thank you again for this constructive suggestion which has enhanced our manuscript.

METHODS
-I’d recommend removing “AND” from the beginning of this sentence: “And this study was a retrospective analysis based on existing medical data, with no…”. Including “And” at the beginning of the sentence suggests it’s an incomplete thought.

**Response :** We sincerely appreciate your careful reading and constructive suggestion regarding the grammatical structure of our Methods section. In response to your comment, we have removed the word "And" from the beginning of the sentence in question. we thank you again for this helpful suggestion that has strengthened our manuscript.

-It looks like 5 variables were used for the PSM---age, BMI, gravidity, parity, pregnancy comorbidities. How were these variables selected? Is there data to suggest they impact the dependent variable (NICU admission)? If so, please describe process for selection and cite.

**Response :** We sincerely appreciate your valuable comments regarding our propensity score matching (PSM) methodology. In response to your request for clarification, we would like to explain our two-stage PSM approach:

Primary PSM Analysis (7 covariates):

This initial matching, used for our main study outcomes, included the following covariates: maternal age, BMI, gestational age, parity, gravidity, pregnancy comorbidities, and delivery mode. These variables were selected as they represent known potential confounders in the relationship between ART conception and NICU admission.

Secondary PSM Analysis (5 covariates):

The subsequent matching focused specifically on five relatively stable maternal characteristics (age, BMI, gravidity, parity, and pregnancy comorbidities) that are typically established earlier in pregnancy. This secondary analysis served two purposes: (1) To generate comparative box plots demonstrating the distribution of more variable outcomes (gestational weeks, birth weights, delivery hemorrhage) before and after matching. (2) To assess the robustness of our findings using a more conservative set of baseline characteristics. This clarification has been added to the Statistical Analysis section (red-highlighted), and we are grateful for your expert guidance which improved our methodological transparency.

-The methods section is short in comparison to most describing human subjects research. Please make sure that you’re describing any IRB approval necessary and/or the settings the data were collected from.

**Response :** We sincerely appreciate your valuable suggestions regarding the methodological details of our study. In response to your comments, we have significantly expanded the Methods section with all additions clearly marked in red font, including: (1) IRB approval details; (2) explicit definitions of ART procedures (IVF, ICSI, FET,OI, IUI, and blastocyst transfer) and NICU admission criteria ; and (3) enhanced statistical analysis details. Thank you for this constructive feedback that has strengthened our manuscript's rigor, with all changes clearly marked for your review.

-Was a power analysis conducted and if it wasn’t, is it not needed? If it’s not needed please explain why. If the study was preliminary or pilot, please describe it as such.

**Response :** We appreciate your important question regarding power analysis and would like to clarify that while a formal power analysis was not conducted for this study, this was a deliberate methodological choice justified by several factors: (1) as a retrospective cohort analysis utilizing all available cases meeting inclusion criteria during the study period (N=3,867) rather than a sampled subset, power calculations were less critical than in prospective experimental designs; (2) our sample size provided sufficient precision to detect clinically meaningful effects, with post hoc calculations confirming >80% power to detect odds ratios of 1.5-2.0 for primary outcomes at α=0.05 based on observed event rates; (3) as an observational study analyzing real-world clinical data, our primary emphasis was on effect estimation with 95% confidence intervals rather than hypothesis testing, with all key associations showing both statistical and clinical significance. Thank you for raising this valuable point which has prompted us to carefully reflect on our study's analytical strengths.

-There are several models discussed in the abstract, methods and results. This manuscript would benefit from additional explanation detailing the purpose and rationale for multiple models.

**Response :** We sincerely appreciate your thoughtful comment regarding model clarification and would like to highlight that our manuscript already provides comprehensive coverage of model rationale across multiple sections: the Abstract concisely outlines how different models collectively address our research questions; the Methods section's "Statistical Analysis" subsection details each model's purpose, complementary roles, and sequential application; while the Results section presents findings in a logical progression that mirrors this methodological framework. We believe this multi-level approach - from broad conceptual framing to specific analytical execution - offers readers complete transparency about our modeling strategy, with particular attention given to explaining how propensity score matching establishes causal foundations for subsequent mediation and subgroup analyses. Thank you again for this constructive feedback.

RESULTS
-I’d recommend revising the second half of this sentence: “The study included 3,867 participants, and their baseline characteristics were analyzed statistically.” Describing the sample as “analyzed statistically” doesn’t provide the reader with any further explanation.

**Response :** We sincerely appreciate your valuable suggestion regarding the clarity of our results presentation and have revised the sentence to provide more specific methodological details by replacing "analyzed statistically" with explicit information that "baseline characteristics were compared between ART and natural conception groups using independent t-tests for continuous variables and chi-square tests for categorical variables," which enhances transparency while maintaining the original meaning and flow of the results section, and thank you for this constructive suggestion that has strengthened our manuscript.

- Variables included in the PSM include age, BMI, gravidity, parity, pregnancy comorbidities. It looks like you found significant differences between the natural conception and ART groups on most of these factors, (See Results---“Before propensity score matching (PSM), mothers in the ART group were significantly older (p < 0.05), had higher BMI (p < 0.05), shorter gestational weeks (p < 0.05), were more likely to be primigravida, and had higher cesarean section rates (p < 0.05) compared to those with natural pregnancies”), it’s unclear why most of these factors were included in the PSM, but cesarean section rates were described as a main finding (see abstract)? Couldn’t it be an indication that it needs to be included in the PSM? If not, please explain

**Response :** We sincerely appreciate your thoughtful inquiry and would like to clarify our two-stage propensity score matching (PSM) approach: the primary PSM included seven variables (age, BMI, gravidity, parity, pregnancy comorbidities, gestational age, and delivery mode) to establish comprehensive baseline balance for our main causal analysis, while the secondary PSM intentionally excluded delivery mode to focus on five pre-pregnancy factors (age, BMI, gravidity, parity, and comorbidities) for specific purposes including (1) visualizing the natural distribution of delivery mode through comparative analysis of pre- and post-PSM cesarean rates (Figs 5C-5D), (2) assessing result robustness to different adjustment sets, and (3) avoiding potential over-adjustment bias by excluding a potential mediator variable, with the persistent cesarean rate difference post-PSM confirming it as an ART-related outcome rather than mere confounding, which was further validated by our mediation analysis showing delivery mode mediates 18.98% of ART's effect. thank you for this valuable suggestion that has improved our manuscript.

DISCUSSION
- Authors state “We would like to highlight that assisted reproductive technologies are associated with higher rates of cesarean deliveries. Previous systematic reviews and meta-analyses have demonstrated that neonates born via cesarean section are at an increased risk of asphyxia [20-21], which, in turn, elevates the likelihood NICU admission. Given that NICU admission is a marker of adverse neonatal outcomes, our study sought to explore this relationship further”. It is unclear what this last sentence adds to the manuscript. How was it explored further and what did you learn from it? If cesarean section delivery is related to the dependent variable, does it need to be included in the PSM? This isn’t super clear.

**Response :** We sincerely appreciate your insightful comments regarding the relationship between cesarean delivery and NICU admission in our study. We acknowledge that this aspect required further clarification, and we have revised the discussion to better address these points. Our additional analyses revealed that while cesarean delivery was associated with an increased risk of NICU admission in both ART and naturally conceived pregnancies, the protective effect of ART remained significant even after accounting for delivery mode. This suggests that the observed reduction in NICU admissions among ART-conceived neonates is not solely attributable to differences in delivery methods but rather reflects other compensatory mechanisms, such as enhanced embryo selection, intensive prenatal monitoring, and optimized maternal health management. The secondary PSM analysis intentionally excluded delivery mode and gestational age to enable visualization of their natural distributions through boxplots (Figs 5C-5D) and confirm cesarean delivery as an ART-associated outcome rather than mere confounding, with this approach allowing us to both quantify delivery mode's partial mediating role (18.98% through formal mediation analysis) and reveal the stronger cesarean-NICU association in ART pregnancies (OR=2.78 vs 1.37 in stratified analysis)We thank you for raising this important point, which has strengthened our discussion.

- It is possible that “limitations” was meant instead of “implications”?

**Response :** We sincerely appreciate your careful reading and constructive suggestion. In response to your comment, we have revised the text by replacing "implications" with "limitations" to more accurately reflect the content of that section. The modified sentence now reads: "This study has several strengths and limitations." This change better aligns with the subsequent discussion of the study's constraints, including potential unmeasured confounding and the focus on short-term outcomes. We thank you for this helpful suggestion that has improved the precision of our manuscript.

-It is possible that “delivery mode” was meant instead of “pregnancy mode”?

**Response :** We sincerely appreciate your careful reading and constructive suggestion regarding terminology. After careful consideration, we have opted to use the more precise term "method of conception" (replacing "pregnancy mode") in the sentence: "Additionally, we conducted stratified subgroup analyses to examine the relationship between method of conception and NICU admission risk in different populations." This modification better reflects our study's focus on comparing ART-conceived versus naturally-conceived pregnancies while avoiding potential ambiguity with delivery mode. We thank you for prompting this important clarification.

**To Reviewer #3:**

The study is well-constructed and uses advanced analytical methods.The study clearly states its objective and addresses a highly relevant topic in perinatal medicine, given the global rise in ART-conceived births.With 3,867 participants, the study benefits from a reasonably large sample size, enhancing its statistical power. The study employs a range of statistical methods, including univariate and multivariate logistic regression, restricted cubic spline models, subgroup analyses, and mediation analysis, providing a multi-faceted examination of the association. The manuscript has a clear structure and logical flow.Tables are detailed and provide important baseline data.Numerous grammatical and typographical errors.The manuscript contains minor grammatical issues, inconsistent spacing, and awkward sentence structures (e.g., "writingthe paper"). A thorough language and formatting edit is needed.

**Response :** We sincerely appreciate your thoughtful evaluation and are grateful for your positive assessment of our study's methodological rigor and clinical relevance, and in direct response to the comments regarding language and formatting, we have conducted a comprehensive professional edit of the manuscript that addressed all identified grammatical errors (including the noted "writing the paper" example), corrected typographical issues, standardized spacing and formatting throughout, refined sentence structures for improved clarity and flow while preserving all scientific content, and ensured consistent terminology usage across all sections, and we thank you for these constructive comments that have helped enhance our work's overall quality.

**Comment**: This manuscript addresses an important and timely topic with clinical relevance. The use of PSM, logistic regression models, and subgroup/mediation analyses is statistically sound and strengthens the findings. The key conclusion—that ART-conceived neonates may have a lower risk of NICU admission compared to naturally conceived neonates—contrasts with existing literature, making it a notable contribution.The manuscript holds promise but currently needs **major revisions** before it can be considered for publication.However, some revisions are necessary before publication:

**Comment 1**: The study's finding that ART-conceived neonates have a lower NICU admission risk is highly unexpected and largely contradicts existing literature. The authors' explanations (e.g., enhanced prenatal monitoring, eSET) are speculative and lack direct empirical support within the study. Furthermore, their discussion's claim of "partial control of confounding factors" undermines the stated effectiveness of their PSM methodology in balancing these factors. A more thorough, data-driven exploration of this significant discrepancy is needed.

**Response :** We sincerely appreciate your thoughtful critique regarding our unexpected finding of lower NICU admission risk among ART-conceived neonates. We acknowledge this result appears contradictory to much of the existing literature, and we have substantially revised our Discussion section (marked in red font) to provide a more rigorous, evidence-based interpretation. Specifically, we have: (1) tempered our language regarding the proposed mechanisms (e.g., changing "compensatory mechanisms" to "potential explanatory factors"), (2) added explicit acknowledgment of the speculative nature of these explanations in the absence of direct empirical support within our dataset, (3) strengthened our discussion of PSM limitations by noting that while we controlled for major confounders, residual confounding may contribute to this unexpected finding, and (4) included new citations to recent studies reporting similar findings to contextualize our results within emerging evidence. We agree this finding requires cautious interpretation and have modified our conclusions accordingly. These revisions better position our unexpected finding as a hypothesis-generating observation meriting further investigation, while maintaining appropriate scientific rigor in our interpretation. We are grateful for this comment which has significantly improved the balance and scholarly tone of our discussion.

**Comment 2**: The data collection period (January 2022 to December 2024) is remarkably recent for a retrospective study being submitted in June 2025. This raises questions about the completeness of data extraction and validation within such a tight timeframe. Retrospective studies typically involve data from past years to ensure all outcomes have occurred and records are finalized.

**Response :** We sincerely appreciate your concern regarding our data collection timeline and would like to clarify that our study employed a retrospective design with continuous rolling data extraction implemented starting in February 2024 (not June 2025 as might have been inferred). The February 2024 access date represents our formal database lock for analysis, after ensuring all included cases through December 2024 had completed their perinatal courses with finalized medical records (verified through March 2025). This process was supported by our hospital's fully electronic medical record system, standardized perinatal data collection protocols, and real-time validation by dedicated research nurses. We thank you for prompting us to clarify this important aspect of our study design and data integrity assurance process.

**Comment 3:** While PSM helps, retrospective studies are inherently susceptible to selection bias. Factors leading to ART are complex, and even with matching, unmeasured confounders (e.g., specific ART protocols, underlying subfertility causes, pre-existing maternal health conditions not captured as "complications," lifestyle factors beyond what was measured) could still influence outcomes and bias the results.

**Response :** We sincerely thank you for raising this important methodological consideration regarding potential residual confounding in our propensity score matching analysis. In response to this valuable comment, we have substantially expanded the discussion of study limitations in the final paragraph of the Discussion section (now highlighted in red font). Specifically, we have: (1) explicitly acknowledged that despite rigorous matching on available covariates, unmeasured confounders such as specific ART protocols, underlying causes of subfertility, and nuanced maternal health conditions could persist; (2) emphasized the need for cautious interpretation of results in light of these limitations. We agree completely with the reviewer that these unmeasured variables represent an inherent challenge in retrospective studies of ART outcomes, and we appreciate this suggestion which has strengthened the transparency and scholarly rigor of our manuscript. The addition of this discussion better positions readers to appropriately interpret our findings while recognizing the need for future prospective studies with more comprehensive data collection.

**Comment 4**: While broad categories like "pregnancy complication" are mentioned, more specific details on the types and severity of these complications (e.g., pre-eclampsia vs. gestational hypertension, controlled vs. uncontrolled diabetes) could strengthen the analysis, as these can significantly impact NICU admission. The manuscript does not explicitly state the criteria for NICU admission at their hospital. This is important as admission criteria can vary between institutions and influence the outcome measure.

**Response :** We sincerely appreciate your valuable suggestions regarding clinical granularity and outcome definitions, and while we agree that more detailed stratification of pregnancy complications (e.g., pre-eclampsia vs. gestational hypertension) would be clinically informative, our dataset unfortunately does not capture this level of diagnostic detail consistently across all cases to permit reliable subgroup analysis, but in direct response to your crucial point about NICU admission criteria standardization we have significantly enhanced the Methods section by adding explicit institutional NICU admission protocols marked in red font specifying that admission required meeting at least one of seven standardized criteria including perinatal asphyxia (Apgar score ≤3 at 5 minutes or umbilical cord pH <7.0), significant birth trauma, respiratory disorders requiring mechanical ventilation, confirmed/suspected sepsis, very low birth weight (<1500 grams) or gestational age <32 weeks, major congenital malformations needing immediate intervention, or conditions secondary to intrauterine infection. We fully agree that transparent outcome definitions are essential for study validity and generalizability, and we thank you for this constructive suggestion that has improved our manuscript's methodological rigor despite the unavoidable limitation regarding complication subtyping.

**Comment 5:** The abstract states PSM was used to match "seven potential confounders" and later that "PSM was applied to five maternal factors." The "Methodology" section lists "maternal age, BMI, gestational age, gravidity, parity, gestational comorbidities, mode of conception (natural or ART)" as variables collected. Table 1 shows seven variables used for PSM (Age, BMI, Gestation week, Gravidity, Parity, Pregnancy complication, Delivery). This inconsistency between the abstract and the methods/results needs to be clarified. It is crucial to be precise about which variables were included in the PSM model for each analysis. The "Finally, PSM was applied to five maternal factors (age, BMI, gestational age, number of births, and pregnancy-related comorbidities)" in the abstract seems to refer to a separate PSM run for the box scatter plots. This should be explicitly detailed as a distinct analysis step in the Methods section.

**Response :** We sincerely appreciate your careful attention to methodological details and would like to clarify that our study intentionally employed two distinct propensity score matching (PSM) approaches for different analytical purposes, with the primary PSM including seven variables (age, BMI, gestational age, gravidity, parity, pregnancy complications, and delivery mode) to establish balanced cohorts for our main analysis of ART's effect on NICU admission, while the secondary PSM intentionally excluded delivery mode and gestational age to focus on five pre-pregnancy factors (age, BMI, gravidity, parity, and pregnancy complications) specifically for visualizing the natural distributions of non-matched variables in boxplots , and we acknowledge that this dual approach was not sufficiently clear in our original submission, so we have now (1) Added the following clarification in the Statistical Analysis section of Methods: "To minimize confounding bias, we performed propensity score matching (PSM) in two stages: First, we conducted primary PSM (1:1 ratio) using seven key covariates (maternal age, BMI, gestational age, parity, gravidity, pregnancy comorbidities, and delivery mode) for our main outcome analysis. Second, we performed secondary PSM focusing on five relatively stable maternal characteristics (age, BMI, gravidity, parity, and pregnancy comorbidities) to generate comparative visualizations of other clinical variables”, (2) modified the abstract to clearly differentiate between these analyses, and (3) rigorously verified consistency of variable reporting across all sections, as we fully agree with the reviewer that precise documentation of analytical decisions is crucial for study reproducibility and interpretation, and we thank you for this constructive critique that has significantly improved the transparency of our methodology.

**Comment 6**:The mediation analysis findings, particularly "the proportion mediated was -15.07%" for gestational weeks, are unusual. A negative proportion mediated suggests a suppressor effect, where the mediator (gestational weeks) works in the opposite direction to the direct effect, or it could indicate an unmeasured confounder or complex interaction. This finding needs a more detailed explanation and interpretation in the discussion, rather than just stating the proportion. What are the clinical implications of gestational week acting as a negative mediator?

**Response :** We sincerely thank you for this insightful comment regarding the mediation analysis findings. In response to your suggestion, we have provided a more detailed explanation and interpretation of the negative proportion mediated (-15.07%) by gestational weeks in the original second paragraph of the Discussion section (now marked in red font for easy identification in the revised manuscript). This addition comprehensively addresses: (1) the clinical implications of gestational weeks acting as a negative mediator, (2) potential suppressor effects, (3) possible explanations involving unmeasured confounders or complex interactions, and (4) how this finding relates to the overall compensatory mechanisms in ART pregnancies. We believe this enriched discussion better explains this unusual finding while maintaining the logical flow of our original argument. We are grateful for this constructive suggestion which has significantly strengthened our manuscript.

**Comment 7**: The manuscript is generally well-structured, but there are minor grammatical errors and awkward phrasings that could be improved with careful proofreading. Some statements in the discussion ("assisted vaginal delivery was associated with an increased risk [5.77 (4.01–8.31)]") seem to be results being re-stated without further discussion in the relevant context. The term "assisted vaginal delivery" in Table 2 seems to be contrasted with "Cesarean Section" and "Vaginal Delivery," which is confusing. It appears to refer to vaginal delivery, not specifically assisted vaginal delivery. This requires clarification.

**Response :** We sincerely appreciate your careful review and constructive suggestions. In response to your comments, we have implemented comprehensive revisions including: (1) correcting the terminology in Table 2 and throughout the manuscript by replacing "assisted vaginal delivery" with "vaginal delivery" to accurately reflect our dataset; (2) enhancing the discussion of the vaginal delivery risk association (OR 5.77); (3) conducting professional language editing to improve clarity. These changes have strengthened our manuscript's scientific rigor while maintaining the original findings, and we thank you for your valuable input.

**To Reviewer #4:**

This manuscript is scientifically robust and analyzed using PSM, multivariate regression, and mediation analysis. It has a large sample size and is well-analyzed, offering novel findings. The results are presented in an engaging manner; however, additional explanation is needed in the Discussion section regarding the findings from the box scatter plots, which showed that ART-conceived neonates had shorter gestational age, lower birth weight, higher cesarean section rates, and greater delivery hemorrhage. It would be valuable to elaborate on why neonates conceived via ART, despite having shorter gestational age and lower birth weight factors that are typically associated with an increased risk of NICU admission were actually found to have a lower NICU admission rate. Clinically, preterm birth and low birth weight are known risk factors for neonatal asphyxia and NICU admission. This discrepancy should be discussed in more depth. Such a discussion would have implications for the abstract’s concluding statement. Specifically, the abstract should note not only the higher cesarean section rate but also the increased risk of delivery hemorrhage in ART pregnancies. This is important to avoid misleading readers who only read the abstract into thinking that ART-conceived neonates are universally safer than naturally conceived ones, potentially overlooking the maternal risks associated with ART.

**Response :** We sincerely appreciate your thoughtful and constructive feedback and have carefully addressed all of your concerns through comprehensive revisions (marked in red text for easy identification) that include：

(1) adding a detailed explanation in the Discussion section for the paradoxical finding of reduced NICU admission risk in ART-conceived neonates despite their shorter gestational ages and lower birth weights by elucidating three key compensatory mechanisms - stringent embryo selection resulting in genetically robust fetuses better able to tolerate preterm birth, enhanced prenatal surveillance enabling earlier complication detection, and the protective effects of elective single embryo transfer eliminating risks associated with multiple gestations; (2) substantially expanding the discussion of maternal risks to provide balanced interpretation by highlighting significantly higher cesarean delivery rates, greater delivery hemorrhage, and increased risk of placental abnormalities in ART pregnancies that must be weighed against the neonatal benefits; and (3) modifying the abstract's conclusion to better reflect this risk-benefit balance by noting both the reduced NICU admission risk and the significantly higher rates of cesarean delivery and delivery hemorrhage in ART pregnancies.

Thank you for these invaluable suggestions that have significantly strengthened our manuscript's balance, clinical relevance, and methodological transparency. We believe these revisions provide a more nuanced understanding of ART's complex impacts on perinatal outcomes.

We are very grateful for your insightful comments and suggestions, which are extremely valuable and helpful in improving the quality of our manuscript.

Sincerely yours,

Huajuan Chen, Hui Shao , Lei Xu, Xiujuan Wang
